# Choroidal neovascularization reduced by targeted drug delivery with cationic liposome-encapsulated paclitaxel or targeted photodynamic therapy with verteporfin encapsulated in cationic liposomes

**Published:** 2013-01-10

**Authors:** Nikolai Gross, Mahdy Ranjbar, Charlotte Evers, Jing Hua, Gottfried Martin, Brita Schulze, Uwe Michaelis, Lutz L. Hansen, Hansjürgen T. Agostini

**Affiliations:** 1 Augenklinik, Universitätsklinikum Freiburg, Freiburg, Germany; 2 MediGene AG, Martinsried, Germany

## Abstract

**Purpose:**

Intravitreal antivascular endothelial growth factor (anti-VEGF) application has revolutionized the treatment of choroidal neovascularization (CNV), a hallmark of wet age-related macular degeneration. However, additional treatment options are desirable as not all CNV lesions respond to anti-VEGF injections. Here, we assessed the feasibility of targeted delivery of cationic liposome-encapsulated paclitaxel (EndoTAG-1) in treating CNV. Furthermore, we investigated whether a new formulation of verteporfin encapsulated in cationic liposomes (CL-VTP) enhances the effect of photodynamic therapy (PDT).

**Methods:**

EndoTAG-1, LipoSPA, and CL-VTP were produced by encapsulating paclitaxel, succinyl-paclitaxel, or verteporfin in cationic liposomes (CL). Mice underwent argon laser coagulations at day 0 (D0) to induce CNV. EndoTAG-1 and LipoSPA were injected into the tail vein at D1, D3, D5, D7, and D9. Taxol, CL, or trehalose buffer alone was injected in control animals. At D10, all animals were perfused with fluorescein isothiocyanate (FITC)-dextran. Flatmounts comprising the retinal pigment epithelium, choroid, and sclera were prepared for quantifying the CNV by measuring the area of lesions perfused with FITC-dextran. For PDT, mice received an injection with CL-VTP or Visudyne at D10. One eye was treated with PDT while the other served as a control. Evaluation of RPE-choroid-scleral and retinal flatmounts was performed at D12, D14, or D17. Perfusion with FITC-dextran and tetramethylrhodamine-5-(and 6)-isothiocyanate-lectin staining was used to distinguish between perfused and non-perfused choroidal vessels.

**Results:**

EndoTAG-1 or LipoSPA significantly reduced CNV size to 15% compared to trehalose controls. The mean CNV area of mice treated with CL was reduced (though not significantly) to about one-half of the value of the trehalose control group. The same was observed for paclitaxel. Thus, the reduction in the CNV size between treatment with CL and treatment with EndoTAG-1 or LipoSPA was 40%, which was not significant. PDT using either CL-VTP or Visudyne reduced CNV size to 65% (D17) of trehalose control size. CNV size was further diminished to 56% with Visudyne and 53% with CL-VTP when PDT was repeated twice. Most importantly, PDT-associated retinal damage was less pronounced using CL-VTP compared to Visudyne.

**Conclusions:**

Systemic intravenous injection of paclitaxel (EndoTAG-1)- or succinyl-paclitaxel (LipoSPA)-loaded CL had a significant antiangiogenic effect in a CNV mouse model. PDT with CL-VTP was as effective as Visudyne in neovascular obliteration but induced less tissue damage. Our data suggest that systemic application of cationic liposome formulations may serve to treat ocular neovascular diseases. This approach may reduce the need for intraocular injections and may benefit patients with neovascular lesions irresponsive to anti-VEGF treatment.

## Introduction

Age-related macular degeneration (AMD) is the most common cause of vision loss in the elderly population in industrialized countries [1,2]. The wet form of AMD is characterized by the pathological growth of choroidal vessels toward the retina, penetrating Bruch’s membrane, and eventually destroying the photoreceptors of the macula, leaving a non-functional scar. This condition was rarely treatable until recently when anti-VEGF treatment became available. The usual application is an intravitreal injection. More specific targeting would be desirable to minimize potential side effects. However, targeted delivery of antiangiogenic drugs in AMD is not available. We demonstrate in this study that cationic liposomes (CL) are a potential tool for clinical use of targeted antiangiogenic therapy in patients with AMD.

CL made from 1,2 dioleoyl-3-trimethylammonium-propane (DOTAP) and 1,2 dioleoyl-sn-glycero-3-phosphocholine (DOPC) but not neutral liposomes [3] bind specifically to activated endothelial cells and are internalized by them [4]. Our previous study demonstrated that CL accumulate in active angiogenic lesions in a murine model of laser-induced choroidal neovascularization (CNV), and CL conjugated with fluorophores such as fluorescein, rhodamine, or indocyanine green can be used for in vivo imaging of the active CNV lesions [5]. Other groups reported similar findings in cancer or lung where neovascularization is involved in the pathogenesis [6,7].

Paclitaxel (the drug is called Taxol) is a substance found in yew, a coniferous tree of the genus *Taxus*. As paclitaxel inhibits cell division, the drug is predominantly used in tumor therapy. In the eye, paclitaxel reduced conjunctival scarring following glaucoma surgery [8] and has been used to treat proliferative vitreoretinopathy [9,10]. Paclitaxel reduced angiogenesis in the mouse corneal micropocket assay [8,11]. The major concerns of systemic use of paclitaxel and derivatives thereof are undesirable side effects due to systemic and non-targeted application. Therefore, using a targeting system to precisely deliver the drug to the acting sites is critical for optimizing efficacy and safety.

The specific binding of CL to sites of active angiogenesis was used as a drug delivery system for antiangiogenic drugs in cancer therapy. EndoTAG-1 is such an anticancer drug with paclitaxel encapsulated in CL. Intravenous administration of EndoTAG-1 results in effective inhibition of tumor growth caused by reduced endothelial cell mitosis, induction of endothelial cell apoptosis, and reduction in functional tumor microcirculation. In addition, less metastatic disease was found in mice with tumors treated with EndoTAG-1 [12-15]. EndoTAG-1 has been tested in clinical phase II trials for treating pancreatic cancer and triple-receptor negative breast cancer [16]. The specificity for sites of active angiogenesis and the antiangiogenic effect make EndoTAG-1 an attractive candidate for treating ocular angiogenic diseases, such as wet AMD.

Photodynamic therapy (PDT) was widely used before VEGF inhibitors became available. It is still performed in cases where VEGF inhibitors do not show improvement, at least in combination with them [17-19]. In PDT, verteporfin is injected into the vein and activated by laser light at 689 nm to produce reactive radicals resulting in occlusion of the vessels at the laser application site. A major improvement in PDT was the encapsulation of verteporfin into neutral liposomes (Visudyne) [20]. In the blood, Visudyne forms a complex with low-density lipoprotein (LDL) that is taken up into proliferating neovascular endothelial cells via their LDL receptors [21] and endocytosis. Therefore, testing whether verteporfin encapsulated in CL shows improved performance in PDT was intriguing.

In this study, we tested whether CL are applicable as a novel drug delivery system that allows highly active and potent drugs to be targeted to the neovascularization site. We hypothesize that such a targeting system should result in at least comparable therapeutic efficacy while reducing the side effects. Especially in an indication such as wet AMD, the side effects of systemically applied drugs have to be viewed more critically than in oncology.

To test this hypothesis in the model of laser-induced CNV in mice, we selected paclitaxel and the paclitaxel prodrug paclitaxel succinate for encapsulation in CL. The resulting drug-containing CL are called EndoTAG-1 and LipoSPA, respectively. The study demonstrates that CL are an option for clinical use of targeted antiangiogenesis therapy in patients with AMD. In addition, we show that CL can be loaded with verteporfin (CL-VTP) resulting in a PDT agent that is as effective as Visudyne but shows fewer defects within the retina.

## Methods

### Liposomes

**Figure 1:**
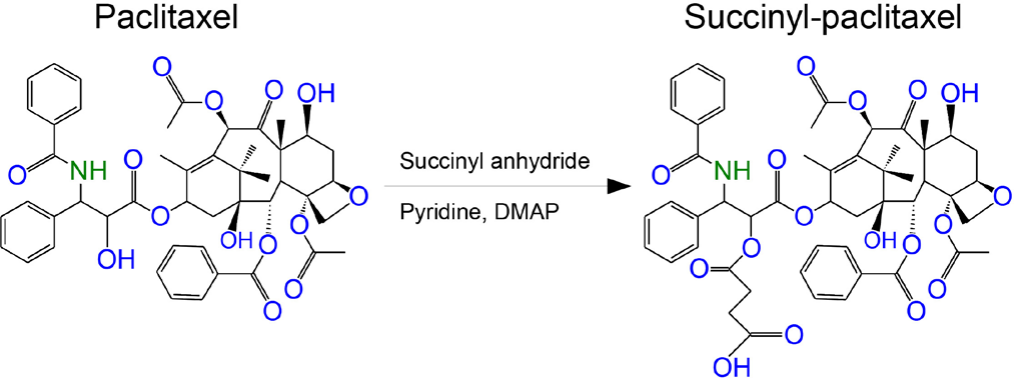
Structures of paclitaxel and succinyl-paclitaxel. Both substances were encapsulated in cationic liposomes. EndoTAG-1 was made from paclitaxel while LipoSPA was made from succinyl-paclitaxel. DMAP=4-(Dimethylamino)pyridine.

CL were loaded with paclitaxel, succinyl-paclitaxel, or verteporfin as detailed below [14] (Figure 1). DOTAP and DOPC (Avanti Polar Lipids, Alabaster, AL) were used for synthesizing the liposomes. Paclitaxel (Synopharm, Barsbuettel, Germany), succinyl-paclitaxel (synthesized in-house, see Figure 1), or verteporfin (Novartis, Nürnberg, Germany) was used for encapsulation into CL. Conventional paclitaxel (PAC, Taxol; Bristol-Myers-Squibb, New York, NY) was diluted in 5% glucose (B. Braun, Melsungen, Germany) and used as a control.

CL with a total lipid content of 20 mM were prepared using the lipid ﬁlm method followed by several cycles of extrusion. For EndoTAG-1, LipoSPA, or CL-VTP, 0.006 mmol paclitaxel, succinyl-paclitaxel, or verteporfin, respectively, was dissolved with 0.1 mmol DOTAP and 0.094 mmol DOPC in 15 ml chloroform (Merck, Darmstadt, Germany). For empty CL, paclitaxel, succinyl-paclitaxel, and verteporfin were omitted.

The respective mixture was gently warmed to 40 °C in a round bottom ﬂask, and the solvent was evaporated under vacuum in a rotary evaporator until a thin lipid ﬁlm was formed. Solvent traces were eliminated by drying the ﬁlm at 5 mbar for 60 min. Multilamellar liposomes formed spontaneously when 10 ml 5% glucose (w/v) was added to the ﬂask. The suspension was left overnight to allow maximal swelling of liposomes. The suspension was then extruded five times in a 10 ml extruder (Northern Lipids, Vancouver, BC, Canada) with a thermobarrel thermostated at 30 °C. The pore size of the polycarbonate membrane (Osmonics, Minnetonka, MN) was 200 nm. The resulting suspension was stored at 4 °C under argon.

### Animals

Ten- to 12-week-old C57BL/6J mice (25–30 g) from Charles River Laboratories (Hamburg, Germany) were used. All animal procedures adhered to the animal care guidelines of the Institute for Laboratory Animal Research (Guide for the Care and Use of Laboratory Animals) in accordance with the ARVO Statement for the Use of Animals in Ophthalmic and Vision Research and were approved by the local animal welfare committee.

### Laser-choroidal neovascularization mouse model

The mouse model of laser-induced CNV was performed as previously described [22]. Mice were anesthetized with an intraperitoneal injection of a ketamine (100 mg/kg) and xylazine (5 mg/kg) mixture. Then 0.5% tropicamide (Pharma Stulln, Stulln, Germany) and 0.5% phenylephrine (Ursapharm, Saarbrücken, Germany) eyedrops were used to dilate pupils. Three laser burns per eye were induced with an argon laser (Visulas 532s, Carl Zeiss Meditec AG, Jena, Germany) with the following settings: 100 ms, 100 µm, and 150 mW. The laser wavelength was 532 nm. A bubble at the laser spot indicated the rupture of Bruch’s membrane. Each group consisted of six to eight mice.

EndoTAG-1, LipoSPA, or control solutions (100 µl) were injected into the tail vein at day 1 (D1) after laser treatment, and injections were repeated at D3, D5, D7, and D9. Taxol and EndoTAG-1 were used at a dosage of 0.5 mg paclitaxel/kg bodyweight.

PDT was performed 10 days after CNV induction. After anesthesia, 100 µl of CL-VTP or Visudyne (Novartis, Basel, Switzerland) containing 72 µg of verteporfin (corresponding to 6 mg/m^2^ body surface) was injected into the tail vein. One min (Visudyne) or 60 min (CL-VTP, CL) later, diode laser treatment was applied to one eye while the other eye remained untreated. Laser settings were 30 s, 1.1 mm, 600 mW/cm^2^, and 689 nm (Coherent Opal Photoactivator; Coherent Deutschland, Dieburg, Germany) according to Odergren et al.’s results [23].

For evaluation, mice were perfused with fluorescein isothiocyanate (FITC)-dextran (50 mg/ml, molecular weight 2000 kDa, Sigma, Taufkirchen, Germany) in 0.9% NaCl. The eyes were excised and fixed for 30 min in 4% buffered formalin. The anterior segments of the eyes and the retinas were removed. In the case of EndoTAG-1 or LipoSPA treatment, the RPE-choroid-sclera complex was flatmounted and examined with epifluorescence microscopy with 480 nm excitation and 510 nm emission filters. In the case of PDT, the RPE-choroid-sclera complex and the retina were flatmounted. The flatmounts were stained in 100 µl of 1 mg/ml tetramethylrhodamine-5-(and 6)-isothiocyanate (TRITC)-lectin (BSI) from Griffonia simplicifolia (Sigma, Taufkirchen, Germany) in 1% Triton X-100, 1 mM CaCl_2_, and 1 mM MgCl_2_ in PBS (137 mM NaCl, 2.7 mM KCl, 10 mM Na_2_HPO_4_, 2 mM KH_2_PO_4_) overnight and investigated with fluorescence microscopy.

**Figure 2:**
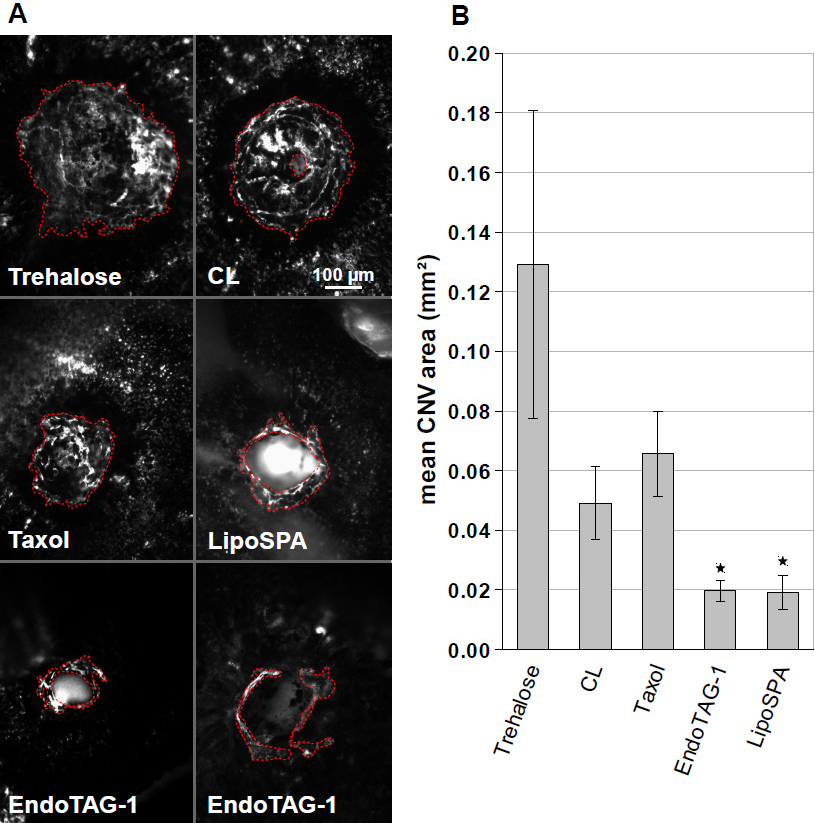
Effect of cationic liposomes on choroidal neovascularization (CNV). CNV was induced by laser coagulation. Test and control substances were injected in the tail vein every second day. Ten days after laser coagulation, mice were perfused with FITC-dextran and scleral flatmounts were prepared. **A**: While trehalose resulted in a large circular neovascularized area (surrounded by a red dashed line), the CNV of cationic liposomes (CL) and Taxol was smaller. EndoTAG-1 and LipoSPA resulted in a small vascularized area around a central hole (surrounded by the inner red dashed line) produced by the laser spot that was not filled in with new vessels as in the control. Sometimes, the center of the hole showed a homogeneous fluorescent signal originating from fluorescent tissues like muscles outside the choroid. **B**: Compared to the trehalose control, all test substances resulted in reduced mean CNV areas. While CL or Taxol reduced the mean CNV area for trehalose to about 50%, EndoTAG-1 or LipoSPA reduced the mean CNV areas further. The differences between trehalose and EndoTAG-1 or LipoSPA were significant (indicated by asterisks). The mean size of up to six laser lesions (three per eye) was calculated for each mouse, and the means and standard errors of each group consisting of six to eight mice are indicated in the graph. P values calculated by ANOVA and Tukey correction: Trehalose/LipoSPA: 0.0121, Trehalose/EntoTAG1: 0.0082.

To quantify the FITC-positive CNV area, the lesions were photographed and outlined with the freehand tool ImageJ (Figure 2A), and the pixels were counted. Uniform central atrophic areas occasionally observed in the center of the CNV were measured in the same way and subtracted from the total area. Statistical analysis of the data was performed in R with unpaired one-way ANOVA (ANOVA) corrected with the Tukey post-test. Significance was assumed for p<0.05.

Retinal vascular defects after PDT were assessed in the FITC-dextran staining. If an area in the size and at the location of a CNV showed interruptions of vessels, the integrity of the vessels was checked in the TRITC-lectin staining. Intact but not perfused areas of vessels were counted and related to the total amount of lasered areas.

### Scanning electron microscopy

Standard procedures were used for scanning electron microscopy. RPE-choroid-scleral flatmounts were prepared 14 days after laser treatment as described. Flatmounts were fixed for three days at 4 °C in a mixture of 3% glutaraldehyde, 2% formalin, and 120 mM sodium cacodylate (pH 7.2). After dehydration in ethanol, specimens were dried by the critical point method with carbon dioxide. Then they were sputtered with gold particles at the surface and imaged with a scanning electron microscope (LEO 435 VP, Zeiss, Oberkochen, Germany).

## Results

### EndoTAG-1 treatment

To induce CNV formation, mice were laser coagulated at D0. They were treated with the respective CL formulations every other day, and RPE-choroid-scleral flatmounts were evaluated at D10. Mice treated with trehalose developed large CNV lesions (Figure 2A). Neovascularization appeared reduced in mice treated with CL or Taxol. Treatment with EndoTAG-1 or LipoSPA resulted in the least neovascularization forming a ring around the laser site that often was not filled in with neovascular material.

**Figure 3:**
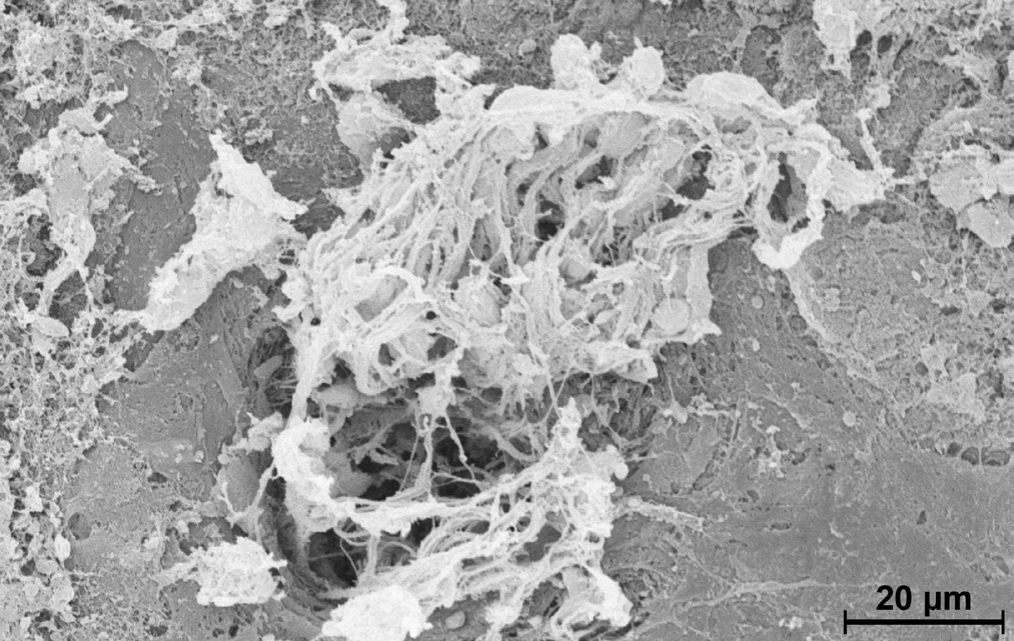
Scanning laser microscopy of choroidal neovascularization (CNV). A laser site 14 days after laser coagulation shows a central hole in the choroid and retinal pigment epithelium (RPE) from which a bundle of vessels is penetrating the retina (retina was removed during preparation).

Quantitative analysis confirmed the morphological results (Figure 2B). Injection of trehalose buffer resulted in a mean CNV area of 0.13 mm^2^. This was reduced to 0.049 mm^2^ by CL or to 0.066 mm^2^ by Taxol. Injection of EndoTAG-1 was associated with a mean CNV area of 0.020 mm^2^, while LipoSPA yielded a mean area of 0.019 mm^2^. The mean CNV areas of EndoTAG-1 or LipoSPA were significantly lower than those of the trehalose control, but they were not significantly lower than those of the unloaded CL or Taxol due to considerable statistical variability. Similarly, the differences of trehalose buffer compared to unloaded CL or Taxol, respectively, were not significant. The results for LipoSPA were close to those of EndoTAG-1 indicating that paclitaxel and the prodrug are equally effective. The three-dimensional morphology of a laser CNV lesion is shown in Figure 3.

### Photodynamic therapy

CNV formation was induced with laser coagulation at D0. PDT was performed at D10 in one eye while the other eye served as a control. After perfusion with FITC-dextran, RPE-choroid-scleral and retinal flatmounts were stained with TRITC-lectin and evaluated by measuring the area of the CNV.

**Figure 4:**
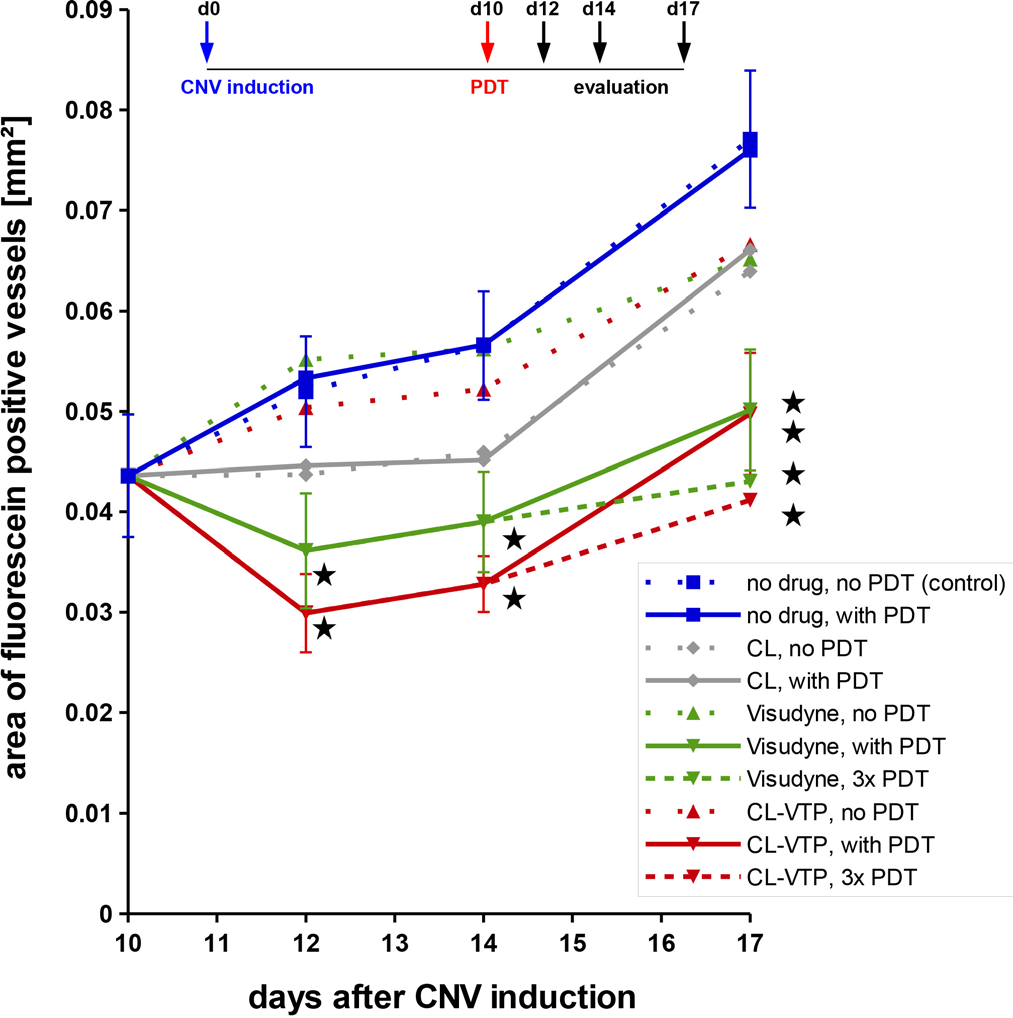
Quantification of photodynamic therapy (PDT) results. Choroidal neovascularization (CNV) was induced at d0 and PDT was performed at d10. The CNV area was measured after perfusion with FITC-dextran. No effects were observed when PDT was omitted (dotted lines). The values for Visudyne or cationic liposomes (CL)-VTP were significantly smaller than those for the controls without drug treatment (solid lines). Repeated PDT even increased the effect (dashed lines). Six to eight mice were used per treatment and time point. Error bars indicate standard errors of the means. Asterisks indicate significant differences compared to the corresponding values without drug treatment as determined with ANOVA. P values calculated by ANOVA and Tukey correction: d12: CL-VTP, with PDT/no drug, with PDT: 0.0159, Visudyne, with PDT/no drug, with PDT: 0.0353, d14: CL-VTP, with PDT/no drug, with PDT: 0.0036, Visudyne, with PDT/no drug, with PDT: 0.0319, d17: CL-VTP, with PDT/no drug, with PDT: 0.0157, Visudyne, with PDT/no drug, with PDT: 0.0173, CL-VTP, 3x PDT/no drug, with PDT: 0.0267, Visudyne, 3x PDT/no drug, with PDT: 0.0447.

Without any drug application, the CNV size reached a mean area of 0.077 mm^2^ at D17 (Figure 4). The CNV size was only slightly reduced when CL, CL-VTP, or Visudyne was applied but diode laser activation was omitted. In contrast, PDT significantly reduced the CNV size to 0.050 mm^2^ for CL-VTP or Visudyne (35% reduction). Repeated PDT at D10, D12, and D14 resulted in further reduced CNV sizes of 0.041 (47% reduction) and 0.043 mm^2^ (44% reduction) for CL-VTP and Visudyne, respectively, at D17. This indicates that CL-VTP is as effective as Visudyne. Interestingly, CL without verteporfin also had a certain beneficial effect while CL-VTP or Visudyne without laser activation had no effect.

**Figure 5:**
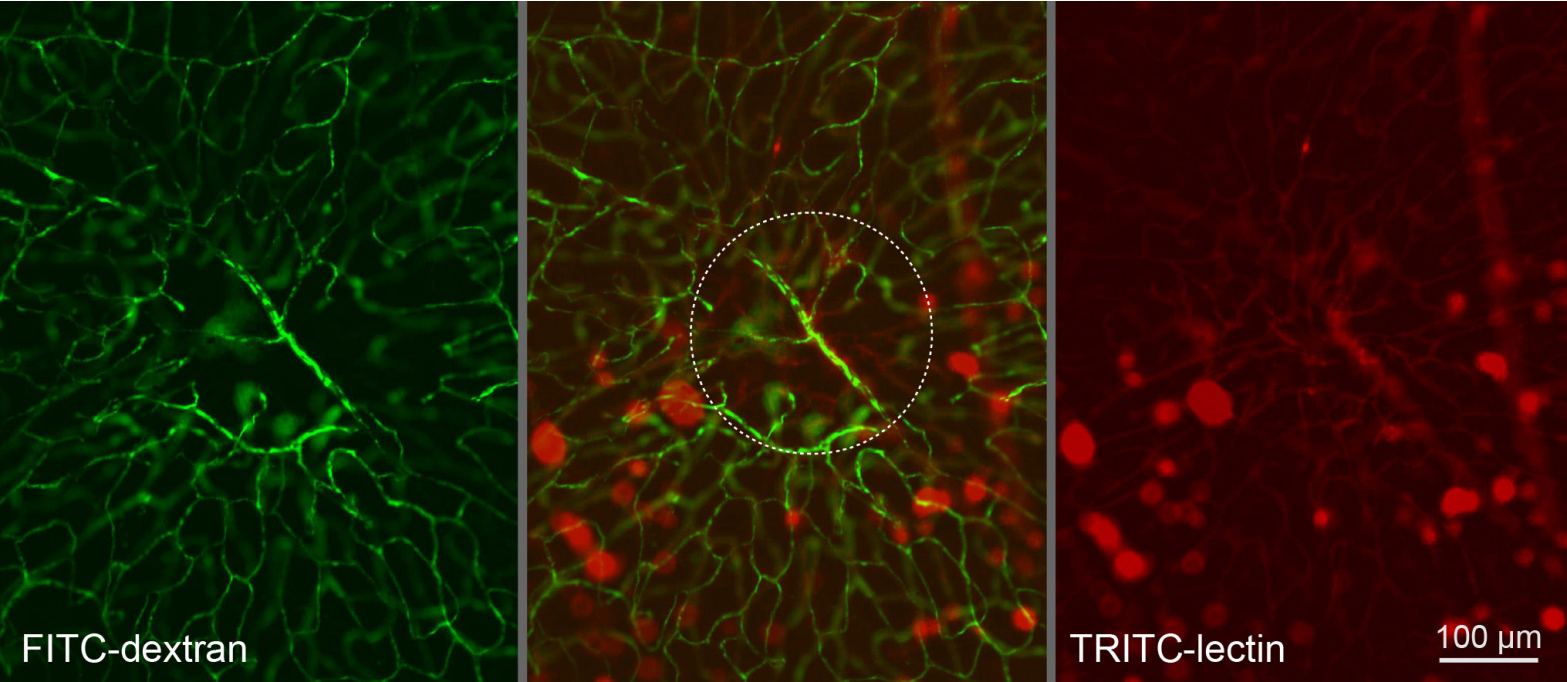
Retinal damage at the laser site at d12. Two days after photodynamic therapy (PDT). The retina was perfused with FITC-dextran and stained with TRITC-lectin. The site of the choroidal neovascularization (CNV) laser treatment in the center of the picture (dashed circle) is not perfused after treatment with Visudyne and PDT though modified capillaries are there as detected with lectin staining. This effect was not found when Visudyne was substituted by cationic liposomes (CL)-VTP or in the controls. Dextran was washed out during lectin staining from the large vessels only but remained in the capillaries. The focus is on the deep retinal vascular network, and the blurred green capillaries are connections to the superficial vascular net that are out of focus.

The laser sites were looked up in the retinal flatmounts. Non-perfused areas in the size of a laser spot showing intact vessels by lectin staining were counted (Figure 5 and Table 1). No or almost no defects of retinal perfusion were found at laser sites if no PDT was performed. Similarly, no defects were found at laser sites if no verteporfin (CL-VTP or Visudyne) was applied. Only the combination of PDT and verteporfin resulted in retinal perfusion defects. These were much more pronounced in the case of Visudyne compared to CL-VTP (Table 1).

**Table 1:** Retinal lesions after laser treatment.

Treatment	Defective/total lesions at d12	Defective/total lesions at d14	Defective/total lesions at d17
no drug, no PDT	0/24 (0%)	0/24 (0%)	0/24 (0%)
no drug, with PDT	1/24 (4,2%)	0/24 (0%)	0/24 (0%)
CL, no PDT	0/12 (0%)	0/12 (0%)	0/12 (0%)
CL, with PDT	0/12 (0%)	0/12 (0%)	0/12 (0%)
Visudyne, no PDT	3/24 (13%)	2/24 (8,3%)	0/24 (0%)
Visudyne, with PDT	15/24 (63%)	13/24 (54%)	9/24 (38%)
Visudyne, 3x PDT		16/21 (76%)	
CL-VTP, no PDT	1/24 (4,2%)	0/24 (0%)	0/24 (0%)
CL-VTP, with PDT	4/24 (17%)	2/24 (8,3%)	0/24 (0%)
CL-VTP, 3x PDT		5/21 (24%)	

Non-perfused areas at laser coagulation sites in the murine retina were detected by FITC-dextran perfusion and compared to the retinal vascular net by TRITC-lectin staining. Numbers of non-perfused sites to total sites and the percentages are given. The treatment was the same as in Figure 4.

## Discussion

Scar formation in wet AMD is a process limited to the fovea and involving neovascularization. As CL are targeting sites of active angiogenesis [4], they might be used as vehicle for drugs to carry them to the site of action. The mouse model of laser-induced CNV was used to investigate this hypothesis.

Paclitaxel interferes with the normal function of microtubules as it stabilizes their structure by binding to β-tubulin. In addition, paclitaxel induces phosphorylation and thus inhibition of Bcl2, which normally inhibits apoptosis. Consequently, paclitaxel showed an antiproliferative effect on endothelial cells (HUVEC) at concentrations higher than 1 pg/ml [15]. Paclitaxel inhibited angiogenesis in the corneal pocket assay of mice [11] or reduced experimental proliferative vitreoretinopathy in rabbits [9,10].

The effect of EndoTAG-1 on microvascular permeability was analyzed in tumors [13,24]. Increased microvascular permeability was found for Taxol or CL, and the effect was even more increased with EndoTAG-1. This result corresponds to the report of reduced tumor growth by Taxol or CL and even more reduced tumor growth by EndoTAG-1 [14]. Increased microvascular permeability caused by EndoTAG-1 is probably due to direct vascular damage, as EndoTAG-1 causes endothelial cell apoptosis and microthrombosis.

In a similar way, the results presented here show that CL or Taxol reduced CNV size (though not significantly), but it was reduced further by EndoTAG-1 or LipoSPA. Although the CNV from control mice filled the entire laser site and extended to the surrounding area, the CNV of mice treated with EndoTAG-1 often preserved a large central hole (Figure 3) surrounded by a small ring of vessels indicating that neovascularization had a much smaller effect. This suggests that EndoTAG-1 might be an effective drug carrier system for treating CNV.

As shown in the present study, the size of the CNV was also reduced after PDT with Visudyne. The 35% reduction in CNV size was similar to that observed in other studies. A 25% reduction in CNV size was shown for PDT with Visudyne at D7 and evaluation at D17 in mice [23]. Similar results were obtained by Ju [25]. In the present study, CL-VTP resulted in an equal effect compared to Visudyne (35% reduction in CNV size) demonstrating that CL-VTP is as effective as Visudyne.

Retinas were investigated for vascular defects at the PDT site. These defects were found almost exclusively in the retinas treated with PDT and Visudyne, while CL-VTP resulted in small defects only. The following two reasons may account for this effect.

Verteporfin coupled to LDL and Visudyne were found in the choroid and RPE extending up to the outer segments of the photoreceptors in rabbits [26]. They remained longest in the RPE. In contrast, CL labeled with fluorescent dyes was specifically accumulated at the sites of CNV [5]. This may be a major reason fewer side effects in retinal vessels were observed in this model.

In the monkey, PDT was effective when applied 20–50 min after Visudyne was injected [27]. Visudyne was shown to be accumulated at the laser CNV sites, and maximal accumulation was reached at 15 min after injection in the rat [28]. However, during the first hour after injection, large amounts of Visudyne remained in circulation being potentially reactive in the retina during PDT laser treatment [29]. In contrast, CL, like other cationic liposomes, are rapidly cleared from circulation within 10 min resulting in a specific accumulation at the CNV after 30–60 min [3-5] that disappeared one day after injection at the latest. This may be the second major reason fewer side effects were observed.

CNV were effectively closed when Visudyne was substituted by verteporfin coupled to Factor VII in the rat [30]. As only 10% of the normal verteporfin concentration was necessary, the side effects detected with histopathologic evaluation were greatly reduced. In general, specific targeting of drugs to the CNV will reduce side effects. In this way, CL could be a major improvement in the PDT technique in angiogenic tissue.

With both therapeutic approaches, we demonstrated that the efficacy of a drug (e.g., paclitaxel or verteporfin) can be enhanced by encapsulation in CL and side effects can be reduced. This agrees with previously published studies. Thus, a carrier system based on cationic liposomes appears to be a universally applicable drug delivery system.
